# Supplementary material for: Production of Bioactive Compounds with Broad Spectrum Bactericidal Action, Bio-Film Inhibition and Antilarval Potential by the Secondary Metabolites of the Endophytic Fungus Cochliobolus sp. APS1 Isolated from the Indian Medicinal Herb Andrographis paniculata
Source: Molecules. 2022 Feb 22;27(5):1459. doi: 10.3390/molecules27051459 (PMC8912084; doi:10.3390/molecules27051459)
Supplement: Supplementary file 1 [file molecules-27-01459-s001.zip › molecules-1562132 supplement.pdf]

Supplementary Table S1. Antibacterial activity (in terms of clear zone inhibition- in mm) of the isolated (from *Andrographis paniculata*) endophytes.

Sl. No.	Name of the endophytic fungi	Name of the bacterial pathogens			
		**MRSA**	**VRSA**	***P. mirabilis***	***E. coli***
1	*Penicillium* sp.	11±0	10±0	11±0.33	11.58±0.33
2	*Rhizopus* sp.	8.33±0.58	7±0	7.33±0.33	6.33±0.33
3	*Fusarium* sp.	9±0.33	8±0.33	9±0.58	10±0
4	*Aspergillus* sp.	09.33±0.33	8.33±0.33	10±0	10.66±0.58
5	*Cochliobolus* sp. APS1	15.66±0.33	15.33±0.33	12±0.58	14.66±0.58

Supplementary Table S2. Antibacterial action of APS1 on different growth medium against MRSA.

Different Fungal Growth Media	Clear zone of Inhibition (mm)
1. Malt extract broth	09±0.33 [a]
2. Czepak Dox broth	11.33±0.33 [b]
3. Potato dextrose broth	15.66±0.33 [c]

Different letters here represent the valid statistical differences (Tukey's multiple HSD test P<0.05).

Supplementary Table S3. Antibacterial action of APS1 in different extraction solvent.

SL. No.	Different Extraction Agent	Clear Zone of Inhibition (mm)
1	Ethyl ether	08±0.33[a]
2	Petroleum ether	15±0[b]
3	n-hexane	14.66±0.33[c]
4	Ethyl acetate	19.33±0.33[d]

Different letters here represent the valid statistical differences (Tuke's multiple HSD test P<0.05).

Supplementary Table S4. Experimental design and results of the Box-Behnken design for the optimization of the antibacterial activity of the fungal isolate *Cochliobolus* sp. APS1.

Run	Independent variables				Response (ZOI)	
	X1: GC (g%)	X2: UC (g%)	X3: MpH	X4: FT (day)	Measured	Predicted
1	0 (10)	0 (0.8)	1 (7)	1 (8)	7.33	7.39
2	0	0	-1 (6)	-1 (6)	11.33	11.40
3	0	0	0 (6.5)	0 (7)	18.33	18.95
4	0	0	-1	1	8.33	8.37
5	0	1 (0.9)	1	0	9.33	9.39
6	1 (9)	0	-1	0	6.00	6.16
7	0	-1 (0.7)	1	0	12.33	12.58
8	0	-1	-1	0	11.66	11.72
9	-1 (8)	-1	0	0	13.00	13.11
10	0	-1	0	1	8.00	8.13
11	0	0	0	0	18.33	18.95
12	1	1	0	0	6.66	6.72
13	0	1	-1	0	8.66	8.79
14	0	-1	0	-1	13.00	13.10
15	-1	1	0	0	10.00	10.13
16	1	0	0	-1	10.66	10.54
17	0	1	0	-1	9.66	9.50
18	-1	0	-1	0	12.00	12.17
19	1	-1	0	0	13.00	13.19
20	0	0	0	0	18.66	18.95
21	-1	0	1	0	12.66	12.76
22	-1	0	0	1	7.66	7.34
23	1	0	1	0	9.00	9.20
24	1	0	0	1	7.00	7.01

25	0	1	0	1	7.33	7.29
26	0	0	1	-1	5.33	5.49
27	-1	0	0	-1	10.33	10.42
28	0	0	0	0	18.66	18.95
29	0	0	0	0	18.33	18.95

GC-Glucose concentration, UC-Urea concentration, MpH-Medium pH, FT-Fermentation time, ZOI-Zone of inhibition (in mm).

Supplementary Table S5. ANOVA for response surface quadratic regression model of antibacterial production by endophytic *Cochliobolus* sp. APS1.

Source	Sum of Squares	DF	Mean of Squares	F-Value	*p*-Value
Model	232.490	14	16.6064	1476.13	0.000
X_1(Glucose conc.)	11.801	1	11.8008	1048.96	0.000
X_2(Urea concentration)	26.403	1	26.4033	2346.96	0.000
X_3(Medium pH)	0.120	1	0.1200	10.67	0.006
X_4(Fermentation time)	21.601	1	21.6008	1920.07	0.000
X_1^2	64.362	1	64.3622	5721.08	0.000
X_2^2	50.401	1	50.4010	4480.09	0.000
X_3^2	44.271	1	44.2713	3935.23	0.000
X_4^2	94.901	1	94.9014	8435.68	0.000
X_1X_2	1.000	1	1.0000	88.89	0.000
X_1X_3	0.003	1	0.0025	0.22	0.645
X_1X_4	0.640	1	0.6400	56.89	0.000
X_2X_3	0.062	1	0.0625	5.56	0.034
X_2X_4	5.522	1	5.5225	490.89	0.000
X_3X_4	0.010	1	0.0100	0.89	0.362
Error	0.158	14	0.0113		
Lack-of-Fit	0.066	11	0.0060	0.20	0.981
Pure Error	0.092	3	0.0306		
Total		28			
R^2	99.93%				
R^2(adj)	99.86%				
R^2(pred)	99.79%				

Supplementary Table S6. Anti-biofilm activity of *Cochliobolus* sp. APS1 culture extract on Gram positive and Gram-negative bacterial pathogens.

Bacterial pathogens	Control (OD570±SD)	Comment	Treated (OD570±SD)	Comment	Inhibition %
B. cereus	1.032±0.012	High biofilm formation	0.108±0.003	Partially inhibited	89.5
B. subtilis	1.198±0.098	High biofilm formation	0.030±0.003	Completely inhibited	97.4
S. aureus	1.007±0.004	High biofilm formation	0.102±0.005	Partially inhibited	89.87
VRSA	0.873±0.010	Intermediate	0.104±0.014	Completely inhibited	88.08
MRSA	0.948±0.041	Intermediate	0.080±0.03	Partially inhibited	91.5
P. mirabilis	0.99±0.009	Intermediate	0.089±0.009	Completely inhibited	91.01
P. aeruginosa	0.886±0.014	Intermediate	0.106±0.006	Partially inhibited	88.03
E. coli	1.006±0.004	High biofilm formation	0.052±0.005	Completely inhibited	94.83
S. flexneri	1.191±0.010	High biofilm formation	0.040±0.002	Completely inhibited	96.64

Supplementary Table S7. Anti-larval activity of EA fraction of *Cochliobolus* sp. APS against *Aedes aegypti* mosquito larvae.

Stage of the larvae	LC_{50} value (µg/ml)	LCL-UCL	LC_{90} value (µg/ml)	LCL-UCL	X^2
II	9.196	0.981-23.109	15.970	2.106-33.792	2.909
III	19.870	3.016-34.160	34.161	6.107-57.163	3.791
IV	25.13	7.001-52.191	48.09	11.797-69.018	2.792

LC_{50}-Lethal concentration of the APS1 extract that kills 50% of the exposed larvae, LC_{90}-Lethal concentration of the APS1 at which 90% of the exposed larvae are eliminated, LCL-Lower confidence limit, UCL-Upper confidence limit, X^2-Chi-square values at $P<0.05$ level.

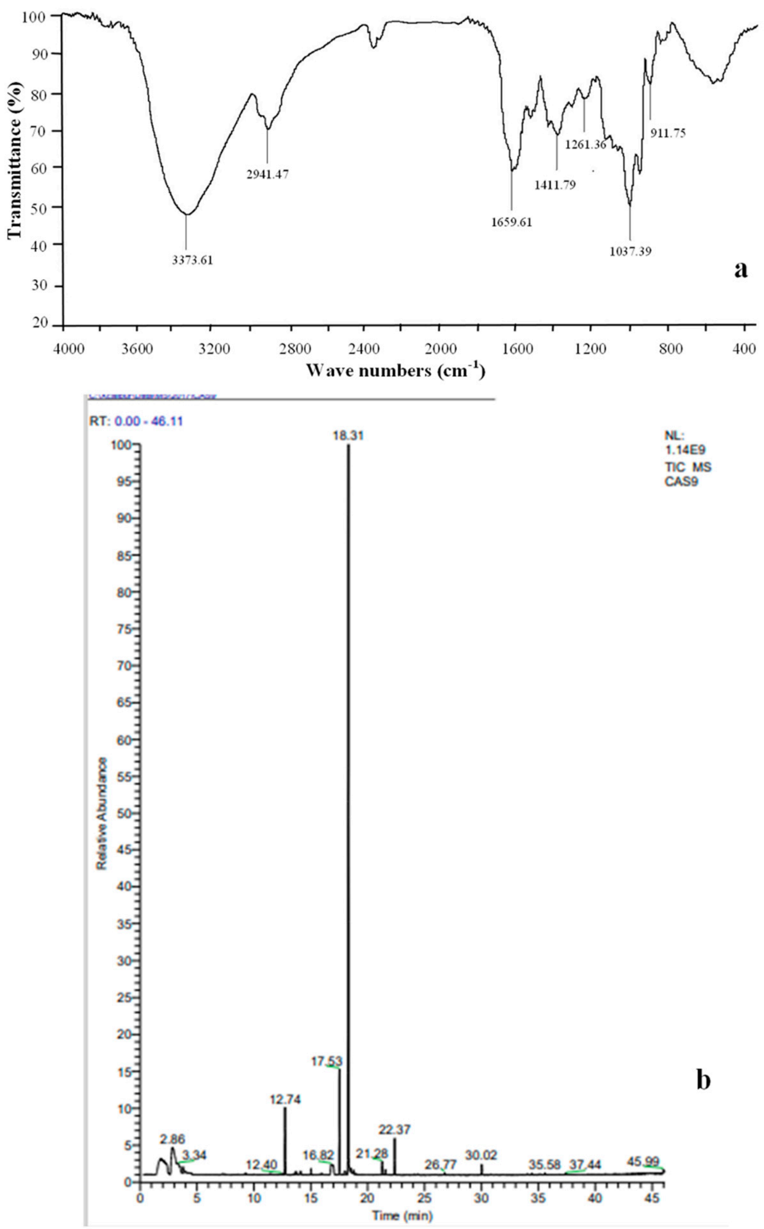

Supplementary Figure S1. (**a**) FT-IR spectra of APS1 EA extract showing the necessary functional groups present in the sample; (**b**) GC-MS spectra of a APS1 EA extract showing major peaks.
